# Room temperature and high response ethanol sensor based on two dimensional hybrid nanostructures of WS_2_/GONRs

**DOI:** 10.1038/s41598-020-71695-3

**Published:** 2020-09-09

**Authors:** Hassan Ahmadvand, Azam Iraji zad, Raheleh Mohammadpour, Seyed Hossein Hosseini-Shokouh, Elham Asadian

**Affiliations:** 1 Institute for Nanoscience and Nanotechnology, Sharif University of Technology, 14588 Tehran, Iran; 2 Department of Physics, Sharif University of Technology, 14588 Tehran, Iran; 3 School of Metallurgy and Materials Engineering, College of Engineering, University of Tehran, Tehran, Iran; 4 Department of Medical Physics and Biomedical Engineering, School of Medicine, Shahid Beheshti University of Medical Sciences, Tehran, Iran

**Keywords:** Nanoscience and technology, Nanoscale materials, Two-dimensional materials

## Abstract

Here in this research, room temperature ethanol and humidity sensors were prepared based on two dimensional (2D) hybrid nanostructures of tungsten di-sulfide (WS_2_) nanosheets and graphene oxide nanoribbons (GONRs) as GOWS. The characterization results based on scanning electron microscopy (SEM), energy dispersive X-ray spectroscopy (ESD), Raman spectroscopy and X-ray diffraction (XRD) analysis confirmed the hybrid formations. Ethanol sensing of drop-casted GOWS films on SiO_2_ substrate indicated increasing in gas response up to 5 and 55 times higher compared to pristine GONRs and WS_2_ films respectively. The sensing performance of GOWS hybrid nanostructures was investigated in different concentrations of WS_2_, and the highest response was about 126.5 at 1 ppm of ethanol in 40% relative humidity (R.H.) for WS_2_/GONRs molar ratio of 10. Flexibility of GOWS was studied on Kapton substrate with bending radius of 1 cm, and the gas response decreased less than 10% after 30th bending cycles. The high response and flexibility of the sensors inspired that GOWS are promising materials for fabrication of wearable gas sensing devices.

## Introduction

Ethanol is one of the most widespread consumable volatile organic compounds in today's industries, medicine, foods, drug and biological applications. Long-term exposure cause irritation of the nose and throat, nausea, fatigue, loss of coordination, damage to the liver, kidneys, and central nervous system, and can cause cancer^1–4^. So, reliable portable room temperature ethanol sensors with a fast and reversible response, along with low cost and low power consumption play significant roles in human health and environmental monitoring. Nowadays, detection of low concentration of harmful gases seems promising through preparation gas sensors based on new 2D nanostructures like transition metal di-chalcogenides layered materials (TMDCs)^5–9^. TMDCs have the general formula MX_2_, where M is a transition metal such as tungsten, and X is a chalcogenide element such as sulfur^10^. They have presented suitable properties to fabricate fast and reversible gas sensor devices due to large surface areas, active edges, availability of surface defects, or vacancies for gas molecules physisorption. Although fast charge transfer process between absorbed molecules and surface of TMDCs speeds up the sensing performance, weak connections between the flakes may cause fluctuating behavior and high electrical resistance and reduce gas response values^11^. Therefore hybrid formations based on relative conductive media and TMDCs can improve stability and gas response as was suggested by Park et al. on rGO/MoS_2_ humidity sensors^12^. Herein, room temperature and flexible resistive ethanol sensors were prepared based on 2D hybrid nanostructures of graphene oxide nanoribbons (GONRs) and WS_2_ nanosheets (as GOWS) by a simple drop-casting method. WS_2_, as one of the most operational and stable members of TMDCs family^13^, is an intrinsic n-type semiconductor with a hexagonal layered structure that its sheets are held together by van der Waals interaction and each layer consists of a slab S–W–S sandwich^14^. GONRs are excellent active and conductive media for gas sensing because of large dangling bonds around the edges and surface functional groups^15,16^. WS_2_ nanosheets were synthesized by chemical vapor transport (CVT) method^17,18^ and GONRs were prepared by unzipping of multi-wall Carbon nanotubes (MWCNTs) as reported by Tour’s group with slight modifications^19^, respectively. GOWSs were prepared in a simple way, the solutions contain of WS_2_ nanosheets and GONRs were mixed with a magnetic stirrer and sonicated at room temperature. Gas sensing properties of GOWS samples were investigated for ethanol vapor and humidity at room temperature (about 25 °C ± 2 °C,) with different molar ratio of WS_2_/GONRs, on SiO_2_ substrate. Morphological and structural analysis of the as-prepared samples were performed using SEM, EDS, and Raman spectroscopy which verified the formation of heterojunction structures between WS_2_ and GONRs. To study the effects of WS_2_ concentration on the gas sensing properties of GOWS, the molar ratio of WS_2_/GONRs (= X) was set to different values and the samples were named as GOWSX with X = 1, 5, 10,15and 20. The heterojunction formations in GOWS samples presented considerable higher and less fluctuating responses. The results on Kapton substrate indicated that GOWS is a competitive material to fabricate flexible and wearable gas sensors.

## Results and discussion

**Figure 1:**
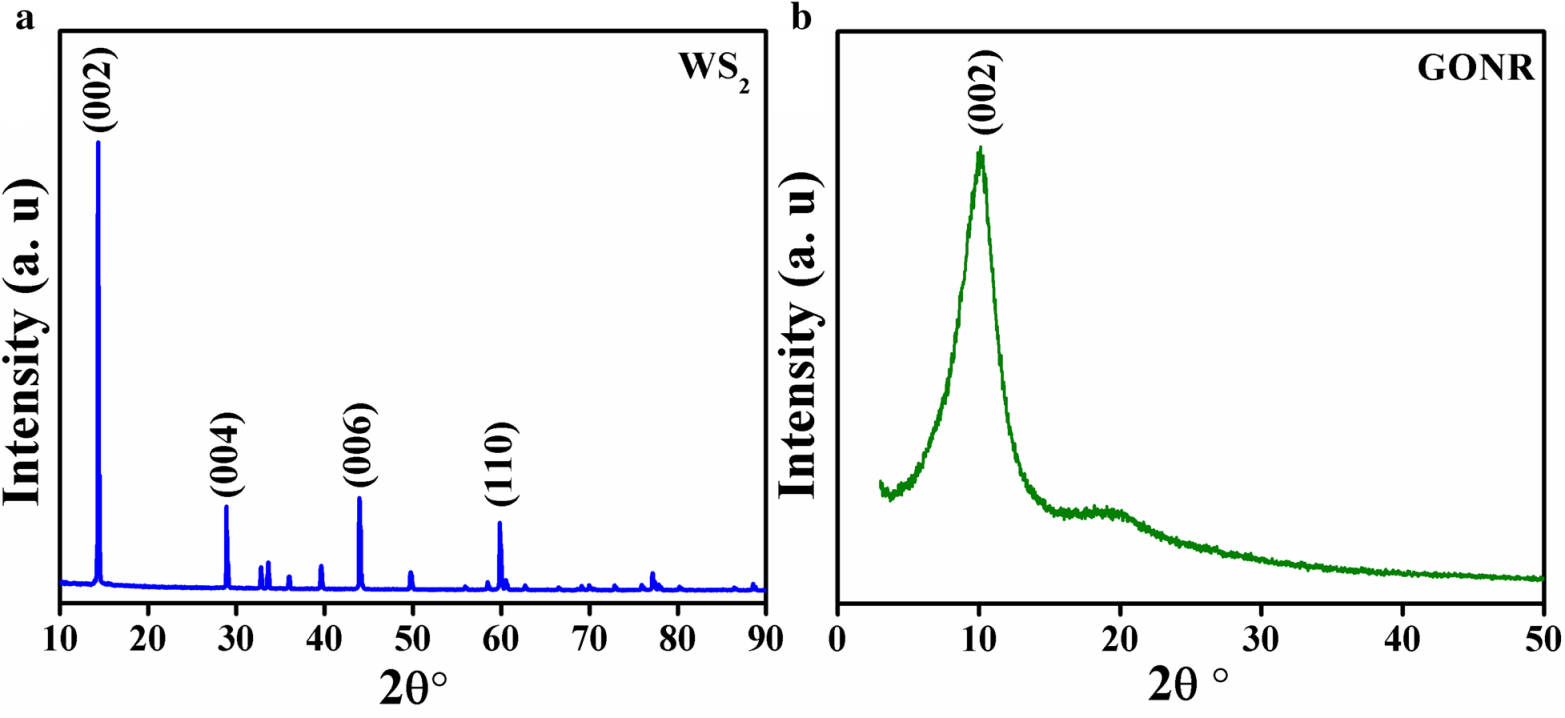
XRD spectra for CVT synthesized WS_2_ (**a**), and GONRs (**b**).

**Figure 2:**
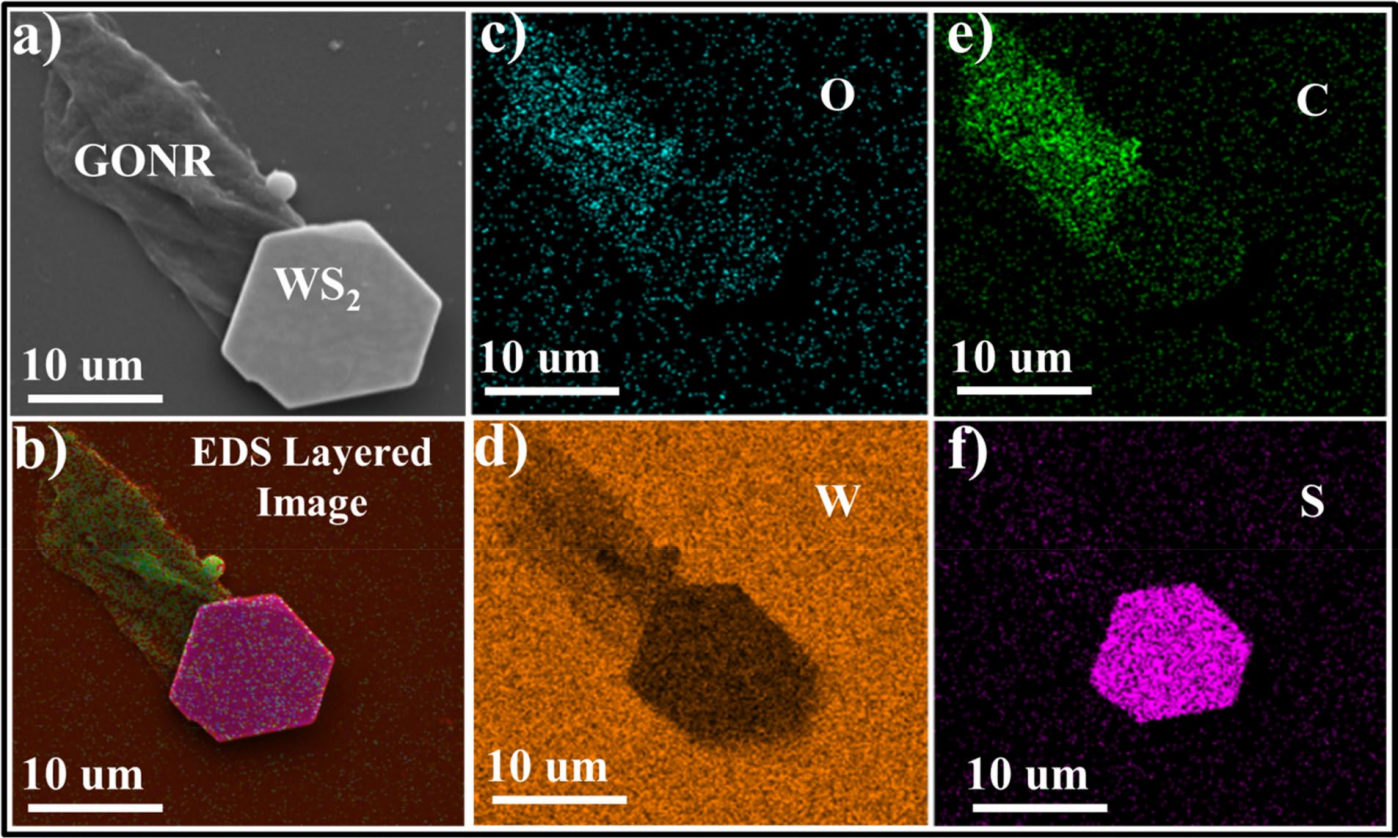
SEM image (**a**), and EDS layered image (**b**) of an attached hexagonal WS_2_ flack with wrinkled GONRs. Elemental map of oxygen (**c**), tungsten (**d**), carbon (**e**), sulfur (**f**), in GOWS.

The synthesized materials were subjected to X-ray powder diffraction analysis (XRD) via a Philips X’pert instrument operating with Cu K_α_ radiation (λ = 1.54 Ǻ) at 40 kV/40 mA diffractometer. The characteristic peaks of WS_2_ and GONRs are appeared in the XRD results as shown in Fig. 1a,b and are in good agreement with those of previous reports^14,20^. Surface morphology and structure of the prepared materials were characterized by means of scanning electron microscope (SEM) and energy dispersive X-ray spectroscopy (EDS) maps. Our microscopic observations showed a uniform dispersion of WS_2_ flakes between nanoribbons in GOWS samples. The SEM image of an attached hexagonal WS_2_ flack with wrinkled GONRs is shown in Fig. 2a. To verify their composition, EDS maps were carried on the sample (Fig. 2b). The elemental distribution images of oxygen, tungsten, carbon, and sulphur are demonstrated in Fig. 2c–f, respectively which indicate the successful formation of WS_2_/GONRs heterojunctions in the prepared samples.

**Figure 3:**
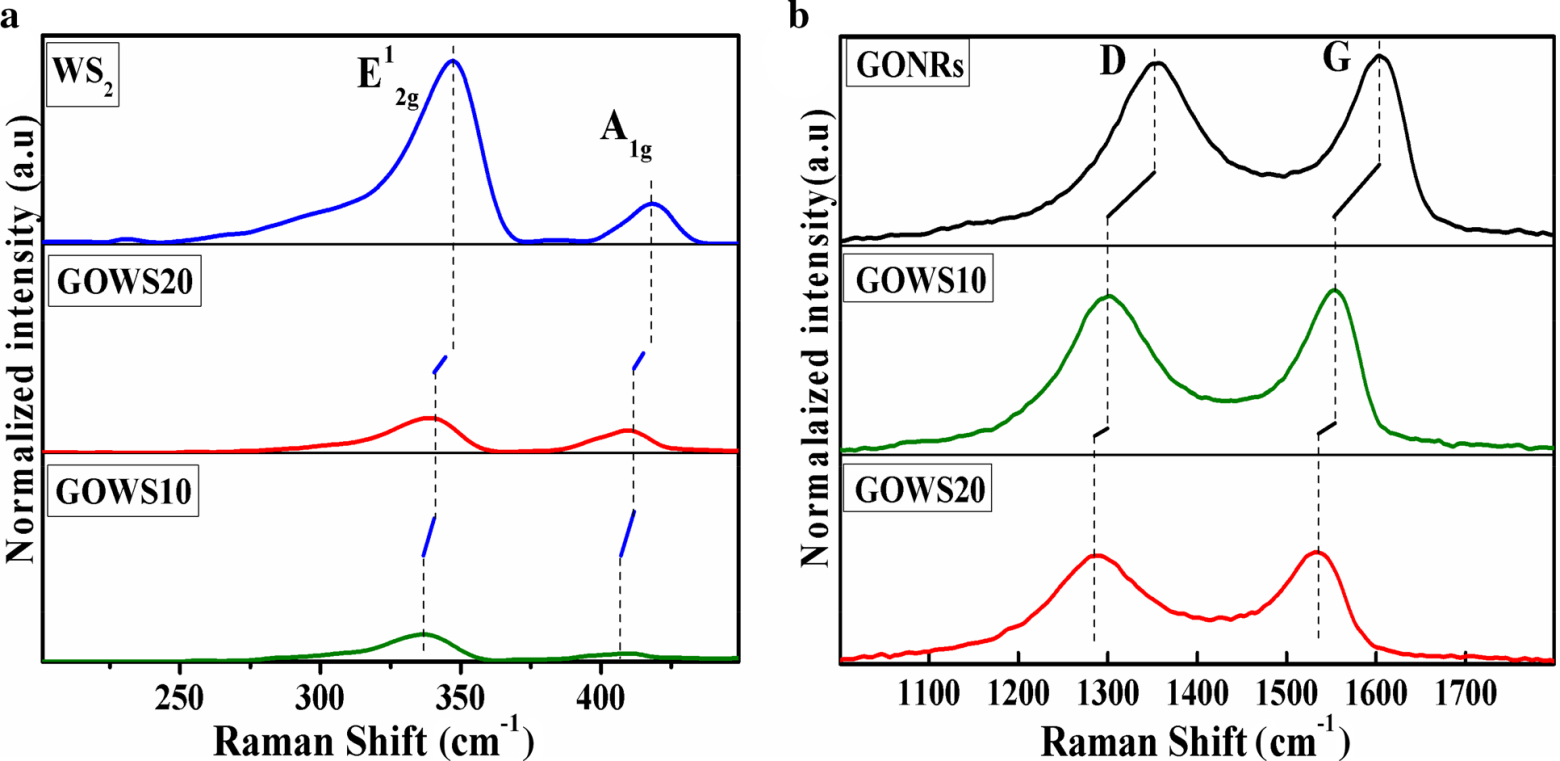
Raman spectra of pristine WS_2_ (**a**) and GONRs (**b**) compared with those of GOWS10 and GOWS20.

In the following, Raman spectroscopy was performed via a Raman spectrometer equipped with an Nd-YAG laser (λ = 532 nm), with a spectral resolution of 1 cm^−1^ (Teksan Raman microscope). The Raman scatterings of GOWS10 and GOWS20 films in comparison to pristine WS_2_ and GONRs are displayed in Fig. 3a,b. The vibrational E_2g_ and A_1g_ modes that are attributed to in-plane and out-of-plane vibrations were appeared at 345.5 cm^−1^ and 416.3 cm^−1^ for pristine WS_2_, respectively^21^. In GOWS20 and GOWS10 samples Raman shifts were observed in E_2g_ from 348.5 to 338.5 and 341.5 cm^−1^, and A_1g_ from 416.3 to 410 and 341.5 cm^−1^, respectively. The characteristic D-band and G-band of GONRs^22^ appeared at 1,360 cm^−1^ and 1,600 cm^−1^, respectively, while the Raman data showed in Fig. 3b represented similar shifts. Therefore the SEM and EDS observations and the shifts in the Raman spectra are evidences for appropriate heterojunctions between WS_2_ and GONRs.

### Gas sensing results

**Figure 4:**
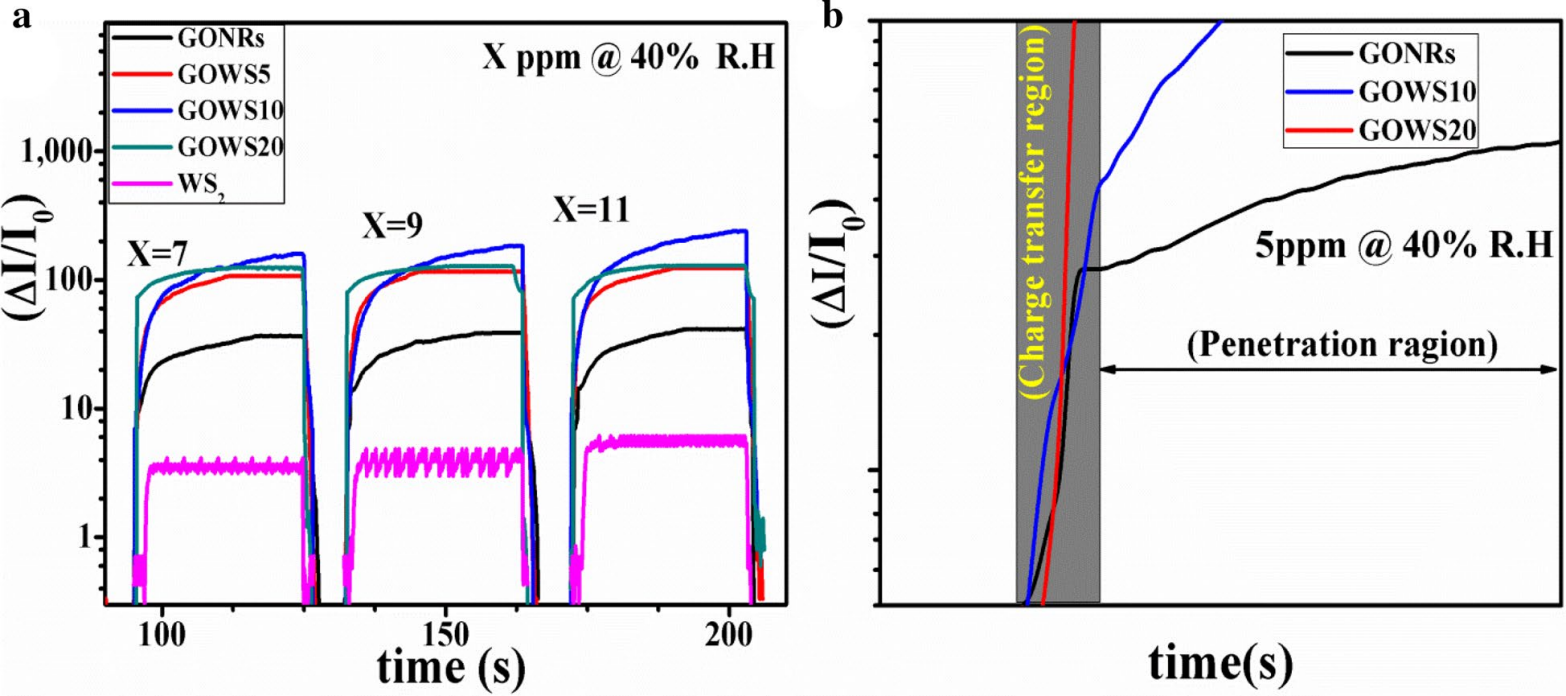
Dynamic response curves of pristine WS_2_, GOWS5, GOWS10, GOWS20 and pristine GONRs for 40% humidity, 7, 9 and 11 ppm of ethanol at room temperature (**a**), an initial rise in response curves for GONRs, GOWS10 and GOWS20 at 5 ppm of ethanol@40% R.H. (**b**).

The sensors were fabricated by drop-casting of equal volume of GOWSs samples, pristine GONRs, and pristine WS_2_ solutions on a SiO_2_ substrate, including sputtered gold interdigitated electrodes with an interspacing of 100 μm and an active area of 10 mm × 10 mm. Ethanol sensing properties of the samples were measured in a breath simulator setup at room temperature. Dynamic gas sensing curves for 7, 9 and 11 ppm of ethanol in a mixture of air with about 40% R.H. were obtained at room temperature and the results are depicted in Fig. 4a. Electrical resistance for WS_2_, GOWS10, and GONRs samples in air was measured about 10 MΩ, 1 MΩ, and 100 KΩ, respectively. The response of the sensors is defined by (I—I_0_)/I_0_ where I_0_ refers to the electrical sensor current in air and I represents the electrical current in the presence of target gas.

**Figure 5:**
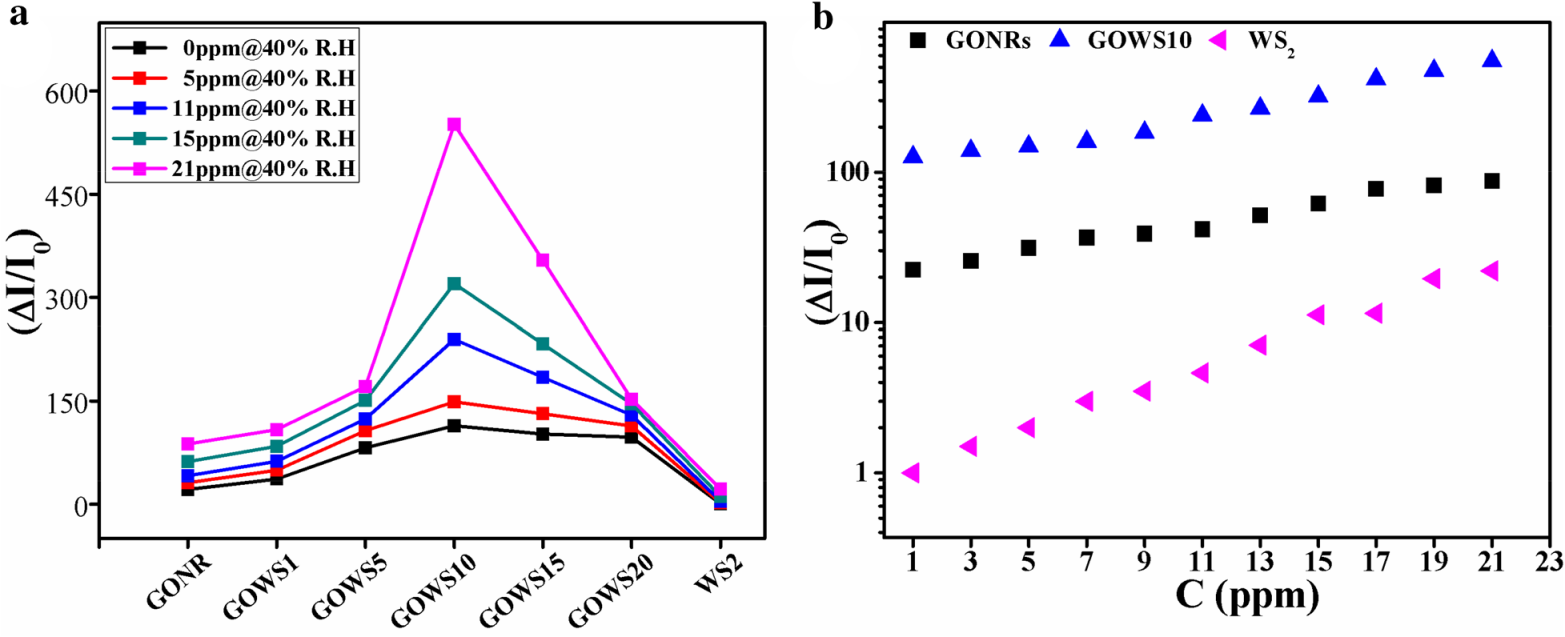
Response values for all samples toward 40% R.H. and 5, 11, 15 and 21 ppm of ethanol (**a**), response values of GONRs, WS_2_ and GOWS10, as a function of ethanol concentrations in a mixture of air with about 40% R.H. (**b**).

GONRs sample indicated higher response and longer rise and recovery times than those of WS_2_, as expected for wrinkle layered structures. Interestingly, the formation of WS_2_/GONRs heterojunctions in GOWS samples resulted in considerable enhancement in the response values for various ethanol concentrations. In fact, graphene oxide nanoribbons enhanced electrical connectivity of the gas sensitive WS_2_ flakes and resulted in a higher and less fluctuating electrical current in GOWS samples. The dynamic curves show two different trends; the fast one is related to charge transfer via water-water and water–ethanol hydrogen-bonding networks, while the slow process is due to the permeation of gas molecules into the inter-layers of GONRs. Figure 4b shows the initial rise in the response curves of GONRs, GOWS10 and GOWS20 at 5 ppm of ethanol@40% R.H. It is rational that sample with higher WS_2_ contents has faster response. Figure 5a displays the response values for all samples toward 40% R.H. and 5, 11, 15 and 21 ppm of ethanol. The results show that GOWS10 have the maximum gas response value compared to others. The response of GONRs, WS_2_ and GOWS10 are presented in Fig. 5b as the function of ethanol concentrations from 1 to 21 ppm in a mixture of 40% R.H.

Two mechanisms have considered for gas sensing; one the hydrogen bond networks formation and the other charge transfer. It is well-known that 2D layered structures of WS_2_ provide high surface sites, such as dangling bonds at the edges, defects and sulfur vacancies, as well as oxygen active sites for physisorption of near-surface molecules^23^. Hence ethanol response could be attributed to charge transfer mechanism^24^ between physisorbed molecules and the active sites. According to previous studies based on density functional theory (DFT) calculations, the physically absorbed ethanol molecules act as electron donor^25,26^ and as result, decrease the electrical resistance of n-type WS_2_. The adsorption energy of the gas molecules is defined by E_(ads)_ = E _(total)_ −E_(WS2)_ − E_(gas)_, where E_(total)_, E_(WS2)_, and E_(gas)_ refer to total energy of the system after gas adsorption on WS_2_, the energy of WS_2_, and the energy of gas molecules, respectively. In general, the charge transfer mechanism could be explained as follows:

$${\text{C}}_{{2}} {\text{H}}_{{5}} {\text{OH}}_{{({\text{gas}})}} + {\text{ V}}_{{({\text{on surface}})}} \to {\text{ C}}_{{2}} {\text{H}}_{{5}} {\text{OH}}_{{({\text{ads}})}} + {\text{ ne}}^{ - }$$

where V _(on surface)_ is the WS_2_ surface vacancies. Upon exposure, the ethanol molecule with low electron affinity serves as electron donor, and transfers its electrons to the conduction band of n-type WS_2_, thus increased electrical conductivity; however, the details in this process are still lack. In addition, humidity response is related to physisorption on the surface active sites and formation of water–water hydrogen bond networks that causes proton hopping as explained in Grotthuss model ^27–29^. Consequently, the network enhances charge transport via the closest physisorbed molecules on WS_2_ flakes. The proton hopping through hydrogen bond networks could be explained as:

$${\text{H}}_{{2}} {\text{O}}^{ + } + {\text{ H}}_{{2}} {\text{O}}^{{}} \to {\text{H}}_{{2}} {\text{O}} + {\text{ H}}_{{2}} {\text{O}}^{ + }$$

**Figure 6:**
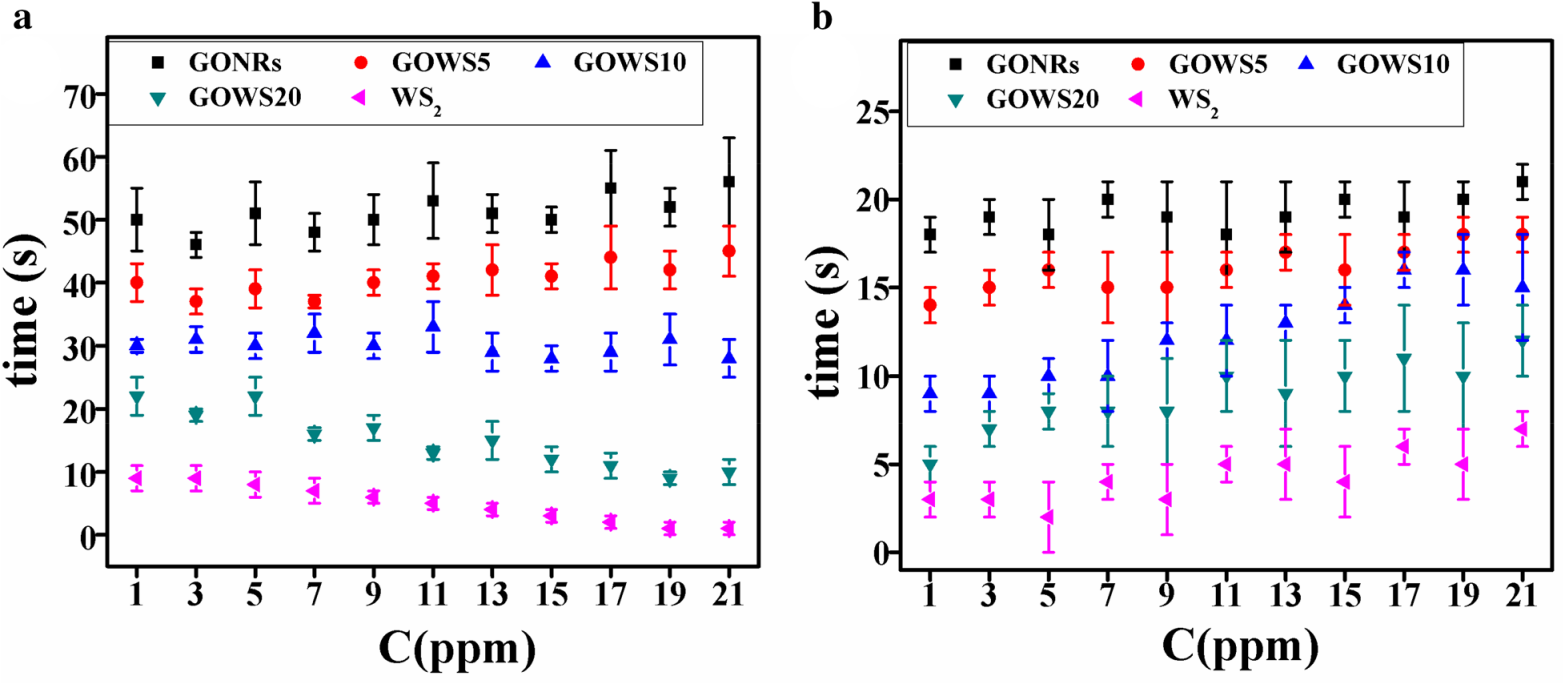
Response (**a**) and recovery (**b**) times of the samples versus ethanol concentration.

Since a higher gas response for WS_2_ sheets was expected if they were electrically connected, GONRs were used to enhance the electrical conductivity. In fact, GONRs with high surface-to-volume ratio, abundant active edges and surface functional groups, is itself a sensitive material for physisorption of molecules^7,30^. Water and ethanol molecules interacted mainly with hydroxyl/carboxyl groups and form hydrogen bond networks^31,32^. In p-type GONR samples seems that the formation of hydrogen bond networks is the dominant mechanism due to the presence huge of GO surface functional groups, and improves the electrical current. So the gas response polarity is positive in p-type samples. It is noteworthy to mention that gradual penetration of the molecules through the graphene oxide inter-layers may results in hydrolyzing the inter-layer functional groups and enhances the ionic conductivity in one hand^33,34^ however, it increases the response times on the other hand. Higher response in the present of the gas mixtures is due to the addition of ethanol–water hydrogen bonds to water–water hydrogen bonds^35–37^. In GOWS hybrid structure, the graphene oxide nanoribbons not only improved the connectivity of WS_2_ nanoflakes but also provided p–n heterojunctions at the interface of p-type GONRs and n-type WS_2_. So, the charge transfer mechanism may reduce the width of the depletion layer at the interfaces of p–n heterojunctions, which is a synergic effect as can be observed in Fig. 5b. The response and recovery times were also investigated as two key factors and the results are shown in Fig. 6a,b, respectively. As mentioned previously, water penetration and desorption are slow process in the case of GONRs which leads to higher response and recovery times in the samples with more values of GONRs along with less variation by increasing the ethanol concentrations. The response and recovery times were about 50 and 20 s for GONRs, and about 28 and 14 s for GOWS10, respectively at 15 ppm of ethanol@40% R.H., while were less than 10 s for WS_2_ samples.

**Figure 7:**
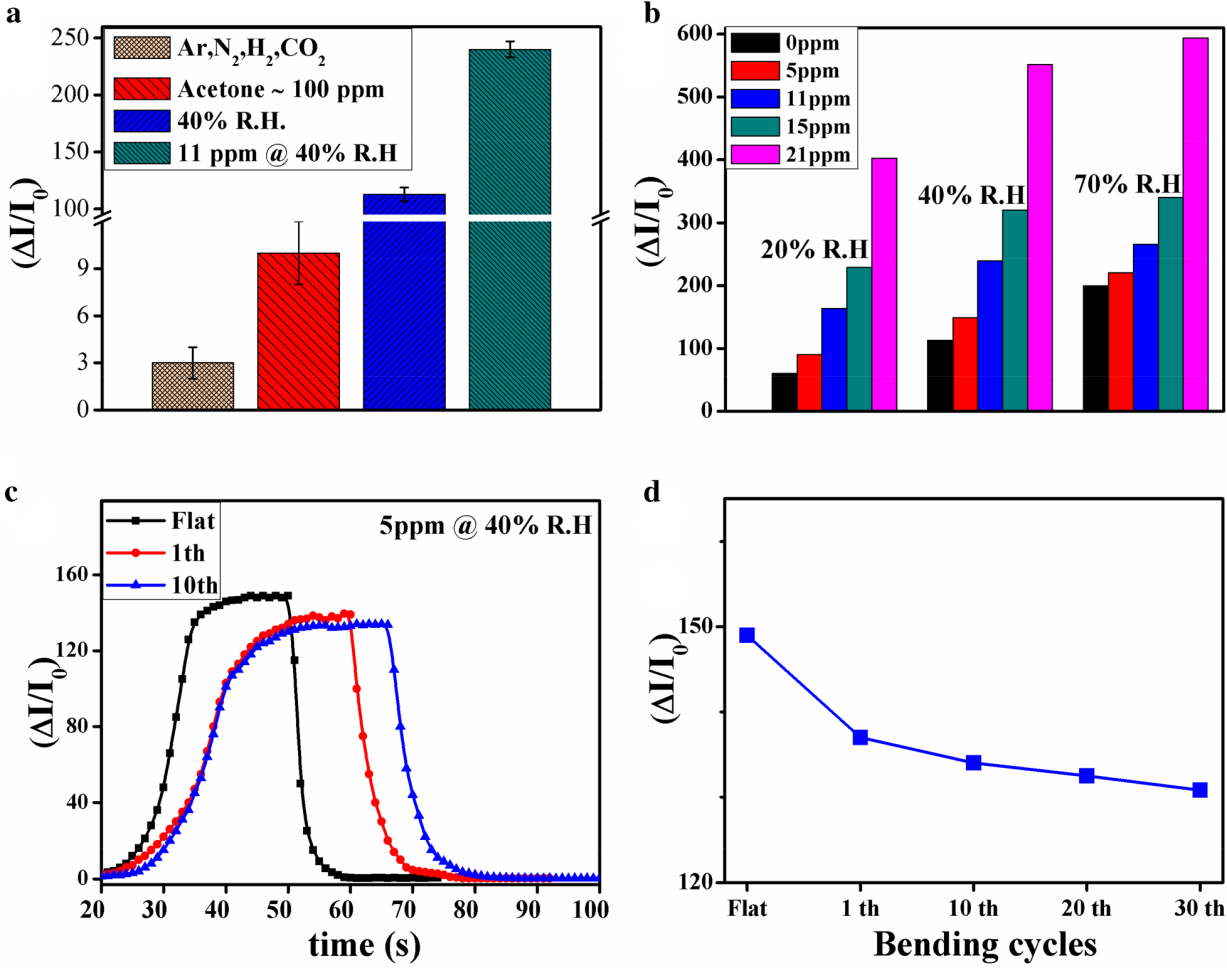
Responses of GOWS10 samples toward H_2_, acetone, dry air (N_2_), Argon, CO_2_, 40% humidity and 11 ppm of ethanol at room temperature (**a**), response values for 5, 11, 15, and 21 ppm of ethanol in mixture of different R.H. (**b**), dynamic response curves of the bent sensors at 5 ppm of ethanol@40% R.H. (**c**), response values for 10th, 20th, and 30th bending cycles test (**d**).

The fast reversibility of WS_2_ may be due to the intrinsic hydrophobic nature of WS_2_^27^ and the large radius of tungsten element that facilitated desorption of gas molecules^26,38^. In the sensor with highest WS_2_ content (i.e. GOWS20), lowest response and recovery times were observed which may be attributed to its high electrical resistance (~ 20 MΩ). Gas selectivity of GOWS10 was measured toward other available gases i.e. H_2_, acetone, dry air, Argon and CO_2_ at room temperature, shown in Fig. 7a, but there was no notable response compared to ethanol and humidity that could be due to the lack of hydrogen bond networks or negligible charge transfer exposed to non-polar or less polar molecules. The response value was about 3 and 10 for dray air and 100 ppm of acetone, while it was 113 and 240 for 40% R.H. and 11 ppm of ethanol @ 40% R., respectively.

To further investigate the role of relative humidity in sensor performance, ethanol response was measured in different relative humidity values. The results have compared in Fig. 7b for 5, 11, 15 and 21 ppm of ethanol in 20%, 40% and 70% R.H. For example, the obtained value was 60 for 20% relative humidity and was 90 for 5 ppm of ethanol @ 20% R.H., respectively. As the relative humidity enhanced up to 70%, the response increased linearly, could be resulting of hydrogen bond networks enhancement. The flexibility of GOWS10 sensor was also studied as an optimized sample coated on Kapton substrate including Au interdigitated electrodes under bending radius of 1 cm. The dynamic response curves of the bent sensor toward 5 ppm of ethanol@40% R.H. are shown in Fig. 7c. As can be clearly seen, before bending (i.e. in the flat mode), the response of sensor was about 149 while upon bending condition it decreased to about 137.5. The response and recovery times increased to 38 and 24 s respectively and the sensor response values decreased less than 8% in the first bending. After 10th bending cycle, the response values further decreased (~ 2.5%) and the response and recovery times increased to about 43 and 29 s respectively. Figure 7d displays the response values for 10th, 20th, and 30th bending cycles. As indicated by the results, the response values, response and recovery times did not show significant changes after 10th bending cycle which turns the proposed sensor to a potential candidate for flexible applications. A summary of the proposed ethanol sensing platforms based on two dimensional composite nanostructures is listed in Table 1 and their results, regarding to detection limit, response value and working temperature as the most important characteristics, are compared with those obtained in the present study.

**Table 1:** A summary of ethanol vapor sensing materials based on 2D nanostructures.

Materials	EtOH (ppm)	Response	Working temperature (°C)	References
SnO_2_@MoS_2_	500	160	280	^42^
ZnO@Graphene	10	8.5	~ 400	^43^
SnO_2_/Graphene	600	38.58	27	^44^
rGO/SnO_2_	50	28.7	170	^45^
PVP/TiS_2_	18	68	RT	^46^
WS_2_/GONRs	1 21	13.5 438.5	RT RT	This work

*EtOH* ethanol vapor concentration, *RT *room temperature.

## Methods

### Preparation of WS_2_ powder

WS_2_ crystals were synthesized by CVT method without employing any transport agent at near atmosphere pressure (100 mbar). In this work, commercial mixed elements; tungsten and sulfur powders (from Merck Ltd., 99/99%) in stoichiometric proportions were located at one end of an evacuated quartz ampule with 20 mm diameter, and length of about 150 mm. The sealed ampule was inserted in a tube furnace for about 8 days before encapsulation at room temperature. This mixture was heated at T_hot_ = 1,323 K (∆T/t = 20 C/min) whereas the other end of the ampule was at lower temperature T_cold_ = 1,123 K. Prior to powder insertion, the quartz ampule was cleaned with piranha, then was rinsed with DI-water and dried at 100 °C to remove contamination.

### Preparation of GONRs

Graphene oxide nanoribbons were synthesized through longitudinal unzipping of MWCNTs^19^. Briefly, 72 mL of H_2_SO_4_ was added to 300 mg MWCNTs in a round bottom flask and stirred for 1 h. Then, 8 mL of phosphoric acid (H_3_PO_4_ 85%) was added to the mixture and allowed to stir for another 15 min before the addition of KMnO_4_ (2.4 g). The reaction mixture was transferred to an oil bath and heated at 65 °C for 2 h until a brownish suspension is obtained. After cooling to room temperature, the solution was poured onto 100 mL of iced DI water containing 10 mL H_2_O_2_ (30%). The resulting light brown colored graphene oxide nanoribbons (GONRs) precipitate was collected by centrifugation (13,000 rpm). Subsequently, the product was washed with DI-water several times until a neutral pH level was achieved.

### Preparation of 2D hybrid nanostructures of WS_2_/GONRs (GOWS)

**Figure 8:**
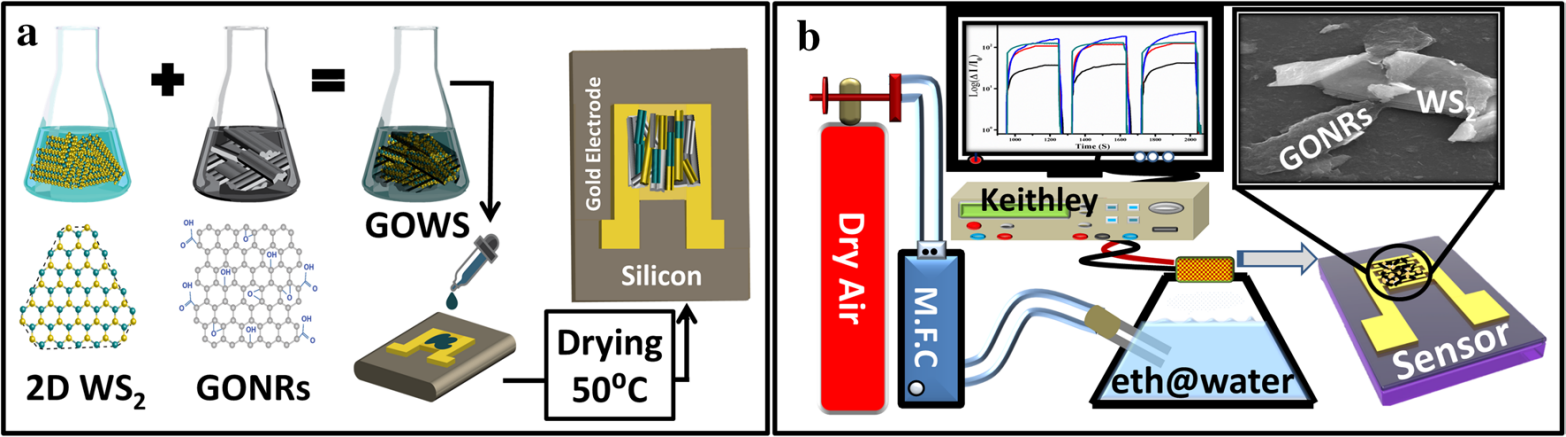
Schematic illustration of mixing process of WS_2_ and GONRs as GOWS hybrid nanostructures and drop-casting method (**a**), schematic representation of ethanol sensing setup (**b**).

The synthesized WS_2_ powder was dispersed in 10 mL of ethanol and sonicated for 3 h at room temperature in order to exfoliate WS_2_ flakes^39,40^. To prepare GOWS hybrid nanostructures and form WS_2_/GONRs heterojunctions two solutions were mixed by using a magnetic stirrer for 1 h and sonicated for 24 h at room temperature^12^. Schematic of GOWS hybrid nanocomposite preparation and drop-casting on the substrate containing gold interdigitated electrodes is shown in Fig. 8a. The molar ratio of WS_2_/GONRs (= X) was set to different values and the samples were named as GOWSX with X = 1, 5, 10,15and 20.

### Sensor fabrication

The ethanol sensing properties of GOWS samples were studied and compared with pristine WS_2_, and GONRs. The sensors were fabricated by drop-casting 40 μL of each solution on a SiO_2_ substrate, including sputtered gold interdigitated electrodes with an interspacing of 100 μm and an active area of 10 mm × 10 mm. After dropping, the substrates spin coated at 250 rpm to form a uniform film, following by drying at 50 °C for 20 min. The flexibility of GOWS was investigated on Kapton substrate with a bending radius of 1 cm.

### Ethanol sensing setup

Ethanol vapor sensing properties of the samples were measured in a breath simulator setup at room temperature. In this setup, the ethanol concentration was controlled by mixing different volume ratios of ethanol (from Merck Ltd., 99/9%) and DI-water (> 18 MOhm-cm). Ethanol vapor was generated by controlling the appropriate inlet dry-air flow to the mixed solution. The ethanol concentration was calculated according to Henry’s law, from 1 to 21 ppm^41^. I–V measurement was performed using Keithely series 6487 Picoammeter as schematically shown in Fig. 8b.

## Summary

Room temperature and high response ethanol and humidity sensors were prepared based on 2D hybrid nanostructures of WS_2_/GONRs by a simple drop-casting method. Morphological and structural analysis of the as-prepared samples verified the formation of heterojunction structures between WS_2_ and GONRs. The GONRs improved electrical connections between WS_2_ nanosheets and the produced p–n junctions upgraded gas response of GOWS hybrid nanostructures. The sensing performance of GOWS was investigated with different molar ratio of WS_2_ to GONRs and GOWS10 exhibited the highest response. The fast performance was observed for GOWS20 sample with response and recovery times of 22 and 5 s at 1 ppm of ethanol@40% R.H. Selectivity of GOWS was studied toward H_2_, Argon, CO, and N_2_ molecules. The flexibility tests were performed based on GOWS10 films on Kapton substrate with bending radius of 1 cm and the results revealed promising potentials for GOWS hybrid nanostructures in wearable sensors applications.

## Data Availability

Derived data supporting the findings of this study are available from the corresponding authors on reasonable request.
